# The interaction of Arabidopsis with *Piriformospora indica* shifts from initial transient stress induced by fungus-released chemical mediators to a mutualistic interaction after physical contact of the two symbionts

**DOI:** 10.1186/s12870-015-0419-3

**Published:** 2015-02-21

**Authors:** Khabat Vahabi, Irena Sherameti, Madhunita Bakshi, Anna Mrozinska, Anatoli Ludwig, Michael Reichelt, Ralf Oelmüller

**Affiliations:** Institute of General Botany and Plant Physiology, Friedrich-Schiller-University Jena, Dornburger Str. 159, 07743 Jena, Germany; Max-Planck Institute for Chemical Ecology, Hans-Knöll-Straße 8, 07745 Jena, Germany

**Keywords:** Microarray, Transcriptome, Defense, Mutualism, Stomata, Reactive oxygen species, Phytohormones

## Abstract

**Background:**

*Piriformospora indica*, an endophytic fungus of Sebacinales, colonizes the roots of many plant species including *Arabidopsis thaliana*. The symbiotic interaction promotes plant performance, growth and resistance/tolerance against abiotic and biotic stress.

**Results:**

We demonstrate that exudated compounds from the fungus activate stress and defense responses in the Arabidopsis roots and shoots before the two partners are in physical contact. They induce stomata closure, stimulate reactive oxygen species (ROS) production, stress-related phytohormone accumulation and activate defense and stress genes in the roots and/or shoots. Once a physical contact is established, the stomata re-open, ROS and phytohormone levels decline, and the number and expression level of defense/stress-related genes decreases.

**Conclusions:**

We propose that exudated compounds from *P. indica* induce stress and defense responses in the host. Root colonization results in the down-regulation of defense responses and the activation of genes involved in promoting plant growth, metabolism and performance.

**Electronic supplementary material:**

The online version of this article (doi:10.1186/s12870-015-0419-3) contains supplementary material, which is available to authorized users.

## Background

The mutualistic interaction between beneficial root-colonizing fungi or bacteria starts with the recognition of both partners before a physical contact is established. Mutual recognition of diffusible signals released by the roots and microbes [arbuscular mycorrhizal (AM), rhizobia-legume root endosymbionts, beneficial endophytes] initiates a signal exchange which prepares the partners for the interaction. Root-derived flavonoids activate the release of factors from the microbes, which induce calcium spiking in root hairs [1]. Downstream of calcium spiking, reprogramming of gene expression in the roots induces mycorrhiza or nodule formation or the establishment of a beneficial mutualistic interaction [2,3]. The symbiotic signals of mycorrhizal fungi, the Myc factors, and those from rhizobial bacteria, Nod factors, are lipo-chitooligosaccharides. They are perceived by lysin-motif (LysM) receptors which induce a signaling pathway leading to either mycorrhiza or nodule formation. Myc factors from *Glomus intraradices* reprogram root gene expression and induce root branching and mycorrhization in *Medicago truncatula* ([4]; and ref. therein). Interestingly, LysM receptors are also involved in the perception of chitooligosaccharides, fungal cell wall compounds that induce defense responses and resistance to pathogens. This raises the question of how plants (legumes) discriminate between beneficial and pathogenic microorganisms (cf. [5]). Furthermore, for the establishment of a mutualistic interaction, the beneficial fungi have to overcome the defense machinery of the host to develop within the host. Kloppholz et al. [6] showed that the AM fungus *G. intraradices* uses the effector protein SP7 to short-circuit the plant defense program. SP7 is secreted and interacts with the pathogenesis-related transcription factor ERF19 in the plant nucleus. *ERF19* is highly induced in roots by the fungal pathogen *Colletotrichum trifolii* as well as by several fungal extracts, but only transiently during mycorrhiza colonization. When constitutively expressed in roots, SP7 leads to higher mycorrhization while reducing the levels of *C. trifolii*-mediated defense responses. Therefore, SP7 is an effector that contributes to develop the biotrophic status of AM fungi in roots by counteracting the plant immune program. These examples show that the symbionts cross-talk *via* chemical mediators which are released into the rhizosphere, and these compounds can be effective prior to the physical contact of the symbionts.

We study the beneficial interaction between the root-colonizing fungus *Piriformospora indica* and the model plant *Arabidopsis thaliana*. The endophyte colonizes the roots of many plant species, and - similar to AM fungi - promotes plant growth, biomass and seed production and confers resistance to abiotic and biotic stress ([7,8]; and references therein). *P. indica* is a member of Sebacinales, grows inter- and intracellularly and forms pear shaped spores, which accumulate within the roots and on the root surface. After the establishment of a beneficial interaction barely any defense or stress genes are activated and no reactive oxygen species (ROS) are produced by the host against *P. indica* [8,9]. Prior to the establishement of a symbiotic interaction and a physical contact between the two partners, *P. indica* releases exudate compounds, which induces appropriate responses in the host. For instance, a fungal compound induces cytoplasmic calcium ([Ca^2+^]_cyt_) elevation in the roots of Arabidopsis and *Nicotiana tabacum*, which is important for establishing the proper host response to the microbe. [Ca^2+^]_cyt_ elevation is followed by a nuclear Ca^2+^ response in the root cells [3]. Rafiqi et al. [10] presented a list of putative effector molecules which were identified in the *P. indica* genome and which might be secreted in order to modulate host cell’s function and structure and to promote microbial growth on plant tissue. Finally, *P. indica* releases small molecular compounds into the medium and the root environment which prevent growth of pathogenic fungi and thereby restrict their growth also in the roots [11].

We have established standardized co-cultivation conditions of *P. indica* and Arabidopsis seedlings on Petri dishes which allow us to investigate the information exchange and the establishment of the mutualistic interaction between the two partners [12]. Here, we report that the seedlings respond to the presence of the fungus as early as two days after co-cultivation although the two organisms have not yet established a physical contact. After six days the hyphae and roots have contact to each other and the first hyphae are detectable within the exodermis of the roots. We report that both roots and leaves respond to the presence of *P. indica* already two days after co-cultivation. The response pattern is quite different four days later, when the hyphae have contact to the roots.

## Results

### Co-cultivation conditions of *P. indica* and Arabidopsis

An agar plaque with *P. indica* mycelium and an Arabidopsis seedling were transferred to a nylon membrane on solidified PNM medium on a Petri dish, with a distance of 3 cm. As control, an agar plaque without fungal hyphae was used (Additional file 1: Figure S1A). Under these co-cultivation conditions, the fungal mycelium and the roots start to grow but they have no contact to each other within the first two days of co-cultivation (Additional file 1: Figures S1B; S2A, B). At this time point, both organisms are separated by at least two cm. Therefore, any communication between the two organisms is only possible *via* exudated soluble compounds into the medium or through the gas phase. After six days of co-cultivation the growing roots and hyphae have reached each other and a physical contact has been established (Additional file 1: Figures S2C, D1, D2). Light and fluorescent microscopical analyses demonstrate that the mycelium penetrates the epidemal layers of the root. Formation of the first fungal spores around the roots becomes also visible (Additional file 1: Figures S2D1, D2). We measured defense and symbiotic responses of the seedlings during the first 14 days of co-cultivation (0, 1, 2, 4, 6, 10, 14 days). After 2 days of co-cultivation, a strong difference in the responses of *P. indica*-exposed and mock-treated control was detectable. After 6 days of co-cultivation, the response pattern was different from that observed at the earlier time point, and did not change much after longer co-cultivation (14 days). We reasoned that the early changes are induced by chemical mediators from the fungus, and that the later changes occur once a physical contact between the two symbionts is established. Therefore, we analysed the response of the roots to the presence of *P. indica* after two and six days of co-cultivation in more details.

### Stomata aperture

**Figure 1:**
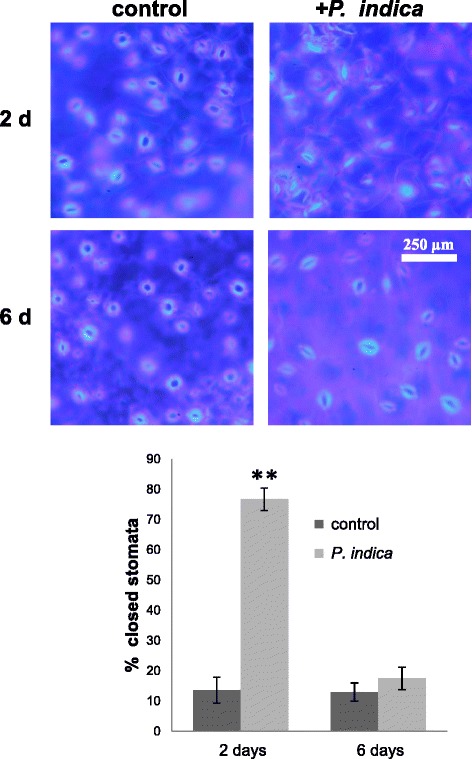
**Epidermis with stomata of Arabidopsis leaves two or six days after exposure of the seedlings to an agar plaque without (control) or with** ***P. indica*** **(+** ***P. indica*** **).** Guard cells were visualized under the fluorescent microscope (450-520 nm) after stained with calcoflour white (the upper level). The lower panel shows the % closed stomata. Based on 3 independent biological experiments with 10 leaves from individual seedlings each. Bars represent SEs. Asterisks indicate significant differences, as determined by Student’s *t*-test (**P < 0.01).

Although a physical contact between the two partners has not yet been established after two days, the leaves of the seedlings respond to the presence of the fungus by closing the stomata (Figure 1). Prior to expose to *P. indica*, 14.6 ± 1.1% of the stomata in the leaves were closed. Almost identical results were obtained for seedlings exposed to an agar plaque without the fungus for either two or six days (two days: 13.9 ± 3.3%; six days: 12.9 ± 3.7%). In contrast, two days after exposure of the seedlings to the *P. indica*-containing plaque, 76.7 ± 2.9% of the stomata were closed. Longer co-cultivation resulted in re-opening of the stomata, and after six days, only 17.5 ± 1.2% of the stomata remained closed (Figure 1). This demonstrates that regulation of stomata opening in the leaves in response to the root-colonizing fungus *P. indica* is a sensitive marker for the interaction of the two partners. To clarify whether the fungal signal(s) is an exudated compound in the medium or a gas, we co-cultivated Arabidopsis seedlings with *P. indica* on split Petri dishes. Exudated compounds from the fungus in the medium cannot reach the roots, while communication *via* gases or volatiles is possible. The number of closed stomata in Arabidopsis seedling was not significantly different two days after co-cultivation of the symbionts on the split Petri dishes compared to the mock-treated control (control: 18.00 ± 1.65%; split Petri dishes: 18.87 ± 2.17%) which excludes gases and volatiles as chemical mediators.

### H_2_O_2_ production

**Figure 2:**
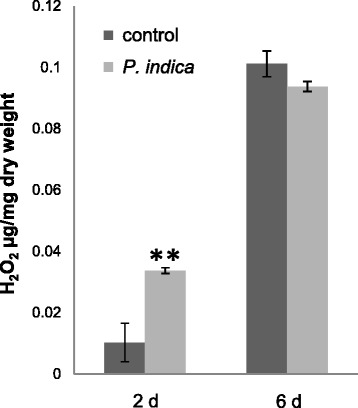
**H**_**2**_ **O**_**2**_ **levels in leaves of** ***A. thaliana*** **seedlings two or six days after exposure to an agar plaque without or with** ***P. indica*** **.** The amount of μg H_2_O_2_ per mg dry tissue was determined as described in METHODS. Based on 3 independent biological experiments with 10 leaves from individual seedlings. Bars represent SEs. Asterisks indicate significant differences, as determined by Student’s *t*-test (**P < 0.01).

High doses of the fungus did not stimulate H_2_O_2_ production in roots and shoots [9] which has been confirmed for roots exposed to *P. indica* for six days (Figure 2). In contrast, two days after co-cultivation, we observed a higher H_2_O_2_ level in the leaves of *P. indica*-exposed seedlings compared to the mock-treated controls (Figure 2). This suggests that exudated compounds from the fungus trigger ROS production, and this stimulatory effect is no longer detectable six days after co-cultivation. Separation of the mycelium from the roots in split Petri dishes prevented the stimulation of H_2_O_2_ production after two days of co-cultivation (control: 0.0033 ± 0.0014 μg/mg dry weight; + *P. indica*: 0.0027 ± 0.0013 μg/mg dry weight), which again supports the involvement of a diffusible compound in the medium.

### Regulation of *NTR2.5* in the leaves in response to *P. indica*

**Figure 3:**
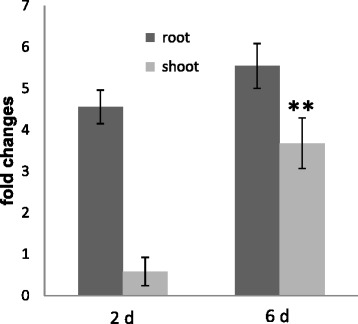
***NRT2.5*** **induction in the roots and shoots of Arabidopsis seedlings which were exposed to** ***P. indica*** **for either two or six days**. The fold change relative to the mock-treatment is presented. Based on 3 independent biological experiments with 3 technical replicates each. Bars are SEs; they represent the sum of the SEs of the individual values. Asterisks indicate significant differences (six day shoot value *vs.* two day shoot value), as determined by Student’s *t*-test (**P < 0.01).

NRT2.5 belongs to the nitrate transporter family and is preferentially, but not exclusively, expressed in leaves. The protein plays an essential role in plant growth promotion by the rhizospheric bacterium strain *Phyllobacterium brassicacearum* STM196 [13,14]. The regulation of its mRNA level in the leaves appears to be very sensitive to signals from the roots. Figure 3 demonstrates that the mRNA level for NRT2.5 in the roots is ~ 4-6-fold up-regulated by *P. indica*, two and six days after co-cultivation. Furthermore, while no significant response can be detected in the leaves two days after co-cultivation, a ~4-fold up-regulation is observed six days after co-cultivation of the seedlings with *P. indica*. This shows that signals from the fungus are transferred to the leaves, although the response is slower than this for stomata closure (Figure 1) and ROS production (Figure 2). The *NRT2.5* mRNA levels in the roots and leaves on split Petri dish experiments were not up-regulated in comparison to the mock-treated controls (data not shown) which again demonstrates that the *NTR2.5* response is mediated by fungus-derived non-gaseous chemical mediators.

### Phytohormone levels in Arabidopsis roots and shoots two and six days after co-cultivation with *P. indica*

**Figure 4:**
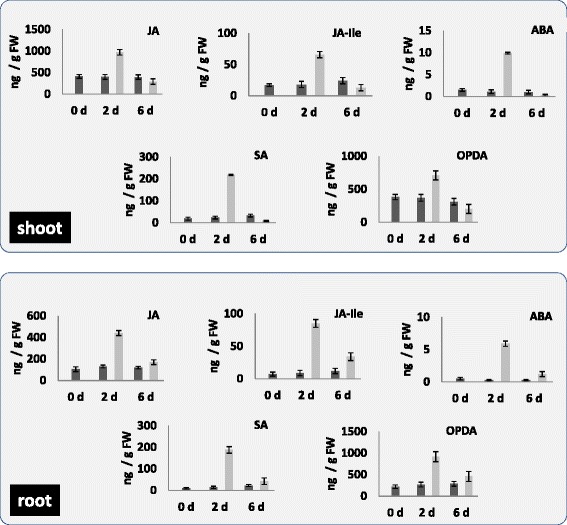
**Phytohormone levels in roots and leaves of Arabidopsis seedlings after exposure to** ***P. indica*** **for two or six days.** The roots and shoots of the seedlings were harvested at day 0, 2 and 6 after exposure to the *P. indica* plug or an agar plug without mycelium. SA, ABA, JA, *cis*-OPDA and JA-Ile levels were determined. The values are means ± SEs of 4 independent biological experiments with 5 replications in each experiment.

Beneficial plant-microbe interactions are associated with changes in phytohormone levels [15-17]. In order to test whether co-cultivation of Arabidopsis roots with *P. indica* affects the phytohormone levels, the amounts of jasmonic acid (JA) and its active form JA-isoleucine (JA-Ile), 12-oxo-phytodienoic acid (OPDA), abscisic acid (ABA) and salicylic acid (SA) were determined in the roots and shoots of seedlings either exposed to *P. indica* or mock-treated. Interestingly, we observed the strongest up-regulation of the phytohormone levels in both roots and shoots two days after co-cultivation. The phytohormone levels decreased significantly in both roots and shoots after six days of co-cultivation (Figure 4). Since the hormones are involved in various types of stress and defense responses, the results indicate that exudated compounds from the fungus induce stress hormones in the roots and systemically also in the leaves. Their level declines as soon as a physical contact between the two organisms is established.

### Transcriptome analyses for Arabidopsis roots two and six days after exposure to *P. indica*

**Figure 5:**
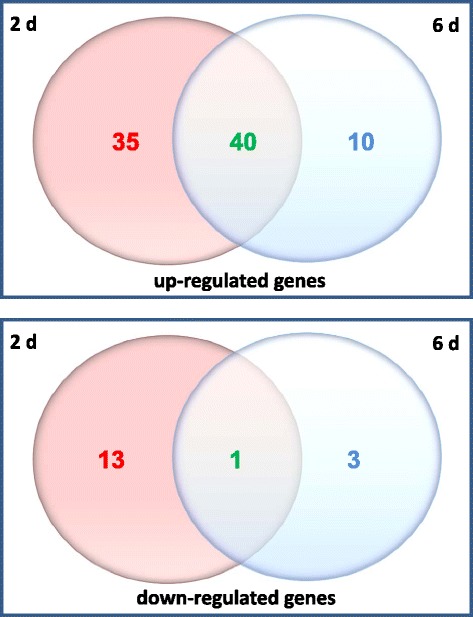
**Venn datagram of the number of genes which are up- or down-regulated in Arabidopsis roots exposed to** ***P. indica*** **for either two or six days.** Numbers of genes regulated only after 2 d of interaction are shown in red colour; those regulated only after 6 d are shown in blue; number of genes regulated at both time points are shown in green. The results are based on 3 independent biological experiments.

**Figure 6:**
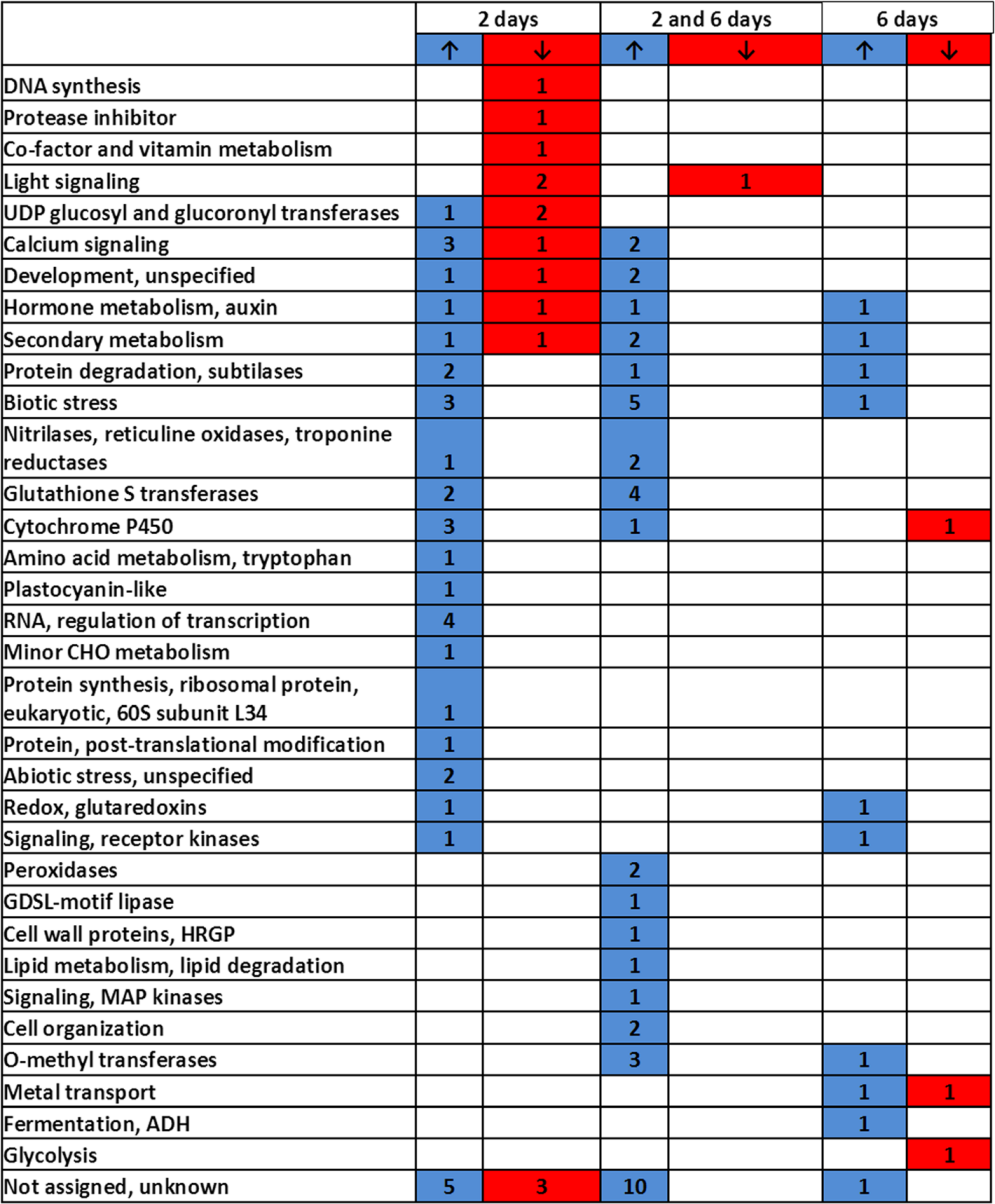
**Number of genes of the MAPMAN categories which are either up-regulated (blue) or down-regulated (red) in Arabidopsis roots 2 or 6 or [** 2 **,** 6 **] days after co-cultivation with** ***P. indica.*** 2 days: genes which are regulated only after 2 days of interaction; 6 days: genes which are regulated only after 6 days of interaction; [2 and 6 days]: common genes which are regulated at both time points. The results are based on 3 independent biological experiments. For detailed information, cf. Additional file 1: Table S1.

Roots exposed to *P. indica* for two and six days were harvested for RNA extraction and expression profiling. Root material exposed to agar plaques served as control. Only genes from *P. indica*-exposed material which showed a > 3-fold difference to the agar control were analysed in this study. The comparative transcriptome analysis [18] uncovered that 75 genes were up-regulated and 14 genes down-regulated after two days, whereas 50 genes were up-regulated and 4 genes down-regulated after six days (Figure 5; Figure 6; Additional file 1: Table S1A, C). Categorization of the genes using the Mapman software revealed a huge difference between the two datasets.

Thirthy-five stress- and defense-related genes are only up-regulated during the early time point of co-cultivation and thus appear to respond to chemical mediators released by the fungus (cf. Discussion; Figure 5; Figure 6; Additional file 1: Table S1A). This includes genes for defense-related cell wall proteins and transcription factors, subtilase At1g32940 [19], a protease inhibitor, chitinase, germin-like protein, PAD3, CYP71B6, galactinol synthase 4, glycosyltransferase 73D1, leucine-rich repeat proteins, glutathione-S-transferases (GST) and glutaredoxin 480. Furthermore, phytohormone-related genes such as *CYP81D8* (At4g37370), *CYSTEINE PROTEINASE1* (At4g36880), *GH3.4* (At1g59500), *TOUCH3* (At2g41100) and those with Ca^2+^-related functions [*CIPK13* (At2g34180) and At4g33050] are also up-regulated two days after co-cultivation (cf. Discussion). In contrast, genes involved in developmental and DNA modifications, such as *HISTONE H1-3*, *PYRIDOXINE BIOSYNTHESIS1.1* and *CML38* are down-regulated.

The number of defense- and stress-related genes is much less after six days of co-cultivation (cf. Discussion).

The majority of the identified genes are regulated by *P. indica* at both time points (Figure 5; Additional file 1: Table S1B). Closer inspection of the expression levels of these genes also confirms a decline in the degree of defense processes from the 2^nd^ to the 6^th^ day after co-cultivation (cf. Discussion). Examples are genes for the root-specific proline-rich extensin At1g26240*,* PHOSPHOLIPASE A 2A (At2g26560), GERMIN-LIKE PROTEIN19, CYP81F2, chitinase At2g43570, the disease resistance protein At2g15120, ENDOPEPTIDASE INHIBITOR1 (At2g43510), the Ca^2+^-binding proteins At5g26920 and At5g39670, the transferase At5g42830, the NAC domain transcription factor JUNGBRUNNEN1, ERD11, ACIREDUCTONE DIOXYGENASE3 and GLUTHATIONE S-TRANSFERASE TAU10 (cf. Discussion). The lower expression level during later stages of co-cultivation indicates that the gene products are less required once a physical contact has been established between the two symbionts.

For 33 randomly chosen genes from the three categories (Additional file 1: Table S1A-C), the microarray results were confirmed by qRT-PCR analyses. Additional file 1: Table S1D demonstrates that most of the results confirmed the microarray data.

To clarify the nature of the fungal signal(s) which modifies the root transcriptome pattern under short term co-cultivation (2 days), we performed co-cultivation experiments on split Petri dishes as described above. The transcriptome pattern of the randomly chosen 33 genes was studied using real-time PCR (Additional file 1: Table S2), but no significant difference was observed to the mock-treated control (Additional file 1: Table S2). This demonstrates again that gases and volatiles do not play a role in changing the gene expression patterns in Arabidopsis roots. Apparently, diffusible compounds released by the hyphae are required for the observed reprogramming of the root transcriptome.

## Discussion

Diffusible compounds released by microbes trigger plant responses before physical cell-to-cell contact occurs [1,20-22]. Several lines of evidence demonstrate that *P. indica* releases compounds which induce defense processes in Arabidopsis roots. The identified genes which are up-regulated after two days of co-cultivation and their role in plant/microbe interaction support this idea. Since the mycelium has not yet reached the roots, plant responses must be induced by either chemical mediators secreted into the medium or gaseous compounds. The split Petri dish experiments support the first possibility, although it cannot be excluded that gaseous compounds also participate in the communication. We also failed to identify major volatile organic compounds which are released into the air in the *P. indica*/Arabidopsis root symbiosis (D. Tholl and R. Oelmüller, unpublished).

Exudate compounds from both fungal mycelium and roots are well characterized mediators of early communication in mycorrhizal symbiosis [23-25]. The exudate from AM fungi induces also nitric oxide (NO) accumulation in *Medicago truncatula* roots [26]. NO is involved in control of stomata closure ([27]; and ref. therein), therefore, fungus-induced and plant-released NO could be involved in the regulation of stomata aperture. The early plant responses in the leaves (stomata closure and ROS production) could be caused by NO of plant origin, which is synthesized in response to chemical mediators released from *P. indica* before a physical contact has been established.

Stomata closure is a typical ABA-mediated stress response, which might be induced by exudated signals from *P. indica*. Many bacterial pathogens invade plants primarily through stomata on the leaf surface. Sawinski et al. [28] showed that microbial invasion is restricted or prevented by stomata closure upon perception of MAMPs, and this represents an important layer of active immunity at the preinvasive level. The signaling pathways leading to stomatal closure triggered by biotic and abiotic stresses employ several common components, such as ROS, Ca^2+^, kinases and hormones, suggesting considerable interaction between MAMP- and ABA-induced stomatal closures. Entry of the foliar pathogen *Pseudomonas syringae* pathovar tomato DC3000 into the plant corpus occurs also through stomatal openings, and consequently a key plant innate immune response is the transient closure of stomata. Kumar et al. [29] showed that root colonization by the rhizobacteria *Bacillus subtilis* FB17 restricts the stomata-mediated pathogen entry of PstDC3000 in Arabidopsis and root binding of FB17 invokes ABA and SA signaling to close the stomata. These results emphasize the importance of rhizospheric processes and environmental conditions as an integral part of the plant innate immune system against foliar bacterial infections, and similar processes may occur in the system described here.

We have previously demonstrated that colonization of Arabidopsis roots by *P. indica* does not result in H_2_O_2_ production [3,8]. Like the regulation of stomata closure, ROS production is fast in response to fungal signals. ROS is also produced during early stages of symbiotic interactions of bacteria and mycorrhizal fungi with roots [30,31]. Here, we demonstrate an early production of ROS before a physical contact between the two symbionts has been established. This is likely initiated by exudated compounds from the fungus. They can function as PAMPs, similar to PAMPs released by pathogenic fungi which activate ROS production *via* activation of the root NADPH oxidase or apoplastic peroxidases, or by gaseous compounds. Our results with split Petri dishes argue against a role of gaseous compounds in this response (Additional file 1: Table S2). These ROS could activate the observed defense responses at the mRNA level, both locally and systemically, two days after co-cultivation of the two symbionts. Fungi also contain NADPH oxidases [32]. *Epichloe festuca*-synthesized ROS regulate hyphal tip growth, thereby restricting growth of the fungus and preventing excessive colonization and host defense gene activation [31,32]. Accumulation of ROS, the oxidative damage to lipids and the membrane electrolyte leakage is lower in AM plants than in non-mycorrhizal plants [33,34], presumably due to the efficient up-regulation of ROS scavenging systems.

Six, but not two days after co-cultivation, we observed the up-regulation of the *NRT2.5* mRNA level in the leaves, indicating a slow root-to shoot signal transduction process in the presence of the fungus. Like *P. indica*, Arabidopsis growth is stimulated by the *Phyllobacterium brassicacearum* STM196 strain, and this is associated with the up-regulation of *NRT2.5* and *NRT2.6* [14]. The *nrt2.5* and *nrt2.6* mutations abolished plant growth and root responses to STM196. Thus, *NRT2.5* and *NRT2.6*, which are preferentially expressed in leaves, play an essential role in plant growth promotion by the rhizospheric bacterium STM196. Members of the *NRT2* family have also been described to be involved in plant defense responses: *NRT2.1* in the priming against *Pseudomonas syringae* pv *tomato* [35] and *NRT2.6* in the resistance against *Erwinia amylovora* [36]. Both genes are required for STM196-induced plant growth promotion, and thus represent new genes in beneficial biotic interactions. Furthermore, these genes participate in a pathway that alters the classically described regulation of shoot - root biomass allocation and root development through the plant nitrogen status. The exact role of these genes in the *P. indica*/Arabidopsis symbiosis remains to be determined, however, *NRT2.5* is a sensitive leaf marker for *P. indica* colonization of the roots.

Phytohormones play important roles in almost all types of plant-microbe interactions. We demonstrate that the defense-related phytohormones JA, Ja-Ile, ABA, SA and OPDA are strongly up-regulated during early phases of co-cultivation of *P. indica* with Arabidopsis roots. Since no physical contact has been established at this time point, their up-regulation must be induced by exudated signals from the fungus (Figure 4). Mukherjee and Ané [37] reported that ethylene inhibits induced symbiotic gene expression and root development in response to germinating spore exudates in mono- and dicots. We observed a quite strong up-regulation of ABA in both roots and leaves in response to secreted fungal compounds (Figure 4). It is consistent with the observed closure of the stomata at this time point. Herrera-Medina et al*.* [38] reported lower colonization of the roots of the ABA-deficient mutant *sitiens* in tomato. Furthermore, the arbuscules were also less developed in the mutant, and both lesions could be restored by exogenous application of ABA to the *sitiens* mutant. It appears that ABA is essential for full AM colonization and arbuscule development (cf. [38]). ABA may down-regulate arbuscular formation directly [39], e.g. by stimulating genes involved in defense and cell wall modification [21], or indirectly by stimulating ethylene production [39]. Garrido et al. [40] showed significant differences in gene expression in mycorrhizal roots of wild-type (WT) and ABA-deficient tomato mutants, and these differences corresponded to the ABA content in the roots. Our data support the important role of ABA in beneficial plant/microbe interactions. Up-regulation of components involved in ABA processes has also been reported by Schäfer et al. [41] in the *P. indica*/barley interaction.

JA, JA-Ile and OPDA are well characterized hormones involved in pathogen attack [42]. Their participation in beneficial plant-microbe interactions is quite controversial (cf. [43]). We observed a strong up-regulation of all these hormonal compounds during early phases of the co-cultivation which is consistent with the observation that JA-regulated stress genes are also up-regulated during the early co-cultivation period. Regvar et al*.* [44], Isayenkov et al*.* [45] and Landgraf et al*.* [46] showed a promotion and Ludwig-Müller et al. [47] a reduction of AM colonization in response to JA or JA-Ile in different systems. Tejeda-Sartorius et al*.* [48] showed that AM colonization was reduced in a JA-deficient tomato mutant [49], and the lesion could be restored by methyl JA application. In contrast, Herrera-Medina et al*.* [50] showed that the JA-insensitive *jai-1* tomato mutant showed increased colonization and the WT tomato was less colonized after methyl JA application. *Nicotiana attenuata* plants silenced for *COI1* expression showed elevated AM colonization [51]. In spite of quite different results, it appears that JA plays a crucial role in beneficial plant-microbe interactions. JA exogenously applied to the growth medium also decreases the number of nodules induced by *Sinorhizobium meliloti* on *Medicago truncatula* roots [52]. JA decreases the responsiveness of Ca^2+^ spiking to Nod factor application and high concentrations of JA inhibited spiking [52], and this might affect root colonization. Application of JA and methyl JA to roots induced the expression of *Nod* genes [53] and the production of Nod factors [54]. This suggests that JA is not exclusively involved in the activation of defense responses. The lower level of JA, JA-Ile and OPDA six days after co-cultivation indicates that these compounds play a less dominant role once the partners have recognized themselves as friends. This resembles reports by Kouchi et al. [55] who showed that during early phases of colonization of *Lotus japonicus* roots by *Mesorhizobium loti* JA-biosynthesis genes are up-regulated. After initiation of nodule formation, they were repressed again.

SA is mainly required for the plant’s defense against biotrophic pathogens (cf. [56]). We observed a strong response in both roots and shoots, but it is not different from the JA, JA-Ile and OPDA responses (Figure 4). An increase in the SA level has also been reported during early stages of AM colonization [57], and this might be important for root colonization by AM fungi [58]. The transient increase in the SA level induces SA-responsive defense genes in *Medicago truncatula* roots at early stages of AM colonization [59], similar to the result described here. Tobacco plants with higher SA levels showed reduced root colonization at early time points, but this effect disappeared during later phases of the interaction [50]. How the defense responses induced by the elevated phytohormone levels are down-regulated when a physical contact between the two symbionts has been established remains to be determined. JA signaling might counteract SA signaling at early stages of the recognition of the two symbionts.

Many genes involved in plant defense are regulated during the co-cultivation of Arabidopsis roots with *P. indica*, however there are clear differences between the early and later time points. Many defense related genes are regulated two and six days after co-cultivation, although their stimulation is lower at the later time point. 35 genes which were up-regulated after 2 days co-cultivation with *P. indica* are stress and defense genes. The germin-like protein 4 (At1g18970) exhibits superoxide dismutase activity and its homologs in barley and wheat are important resistance component against *Blumeria graminis* [59]. The defense-related *WRKY54* [60], *WRKY70* (At3g56400) and *MYB51* (At1g18570) transcription factor genes are involved in basal resistance, stress tolerance [60] or secondary metabolite synthesis [61]. The oxygenic stress-inducible aspartyl protease At3g59080 [62], the HOPZ-ACTIVATED RESISTANCE1 leucine-rich repeat protein (ZAR1, At3g50950) [63], the protease YLS5, the leucine-rich repeat protein kinase At1g51890, the VQ motif protein At4g20000, the WD40 protein (At5g42010, TAIR homepage) and PAD3 (At3g26830, CYP71B15) for camalexin biosynthesis (cf. [64]) participate in different aspects of plant immunity or are induced by pathogen treatments. Several glutathione-S-transferase (GST) genes are also up-regulated at the early time point of interaction. *GSTF3* (At2g02930) responds to *Fusarium sporotrichioides* infection [65] and *GSTL1* (At5g02780) to a wide range of chemicals and abiotic stress treatments [66]. *GST2*, a Ca^2+^-ATPase (At3g63380) is activated by fungal and nematode stimuli and stress (TAIR homepage). Phytohormone-related genes are also up-regulated by chemical mediators from *P. indica*. The antranilate synthase subunit α1 is important for JA-mediated regulation of auxin biosynthesis and transport during lateral root formation [67], GH3.4 (At1g59500) plays an important role in auxin homeostasis [68], the JA-regulated *CYP81D8* (At4g37370) product is involved in phenylpropanoid biosynthesis [69,70], *CYSTEINE PROTEINASE1* (At4g36880) responds to gibberellin [71], and *TOUCH3* (At2g41100) to SA [72,73]. We conclude that many genes which were up-regulated in response to the fungal exudates, code for defense and stress proteins, compounds involved in signaling leading to defense gene activation or control phytohormone homeostasis.

14 genes which are down-regulated two days after co-cultivation with *P. indica* are involved in developmental processes and DNA metabolism. *HISTONE H1-3* (At2g18050) encodes a linker histone protein whose expression is stimulated by dehydration and ABA [74]. *PYRIDOXINE BIOSYNTHESIS1.1* (At2g38230) controls plant growth, development and stress tolerance [75]. *At4g12550* is an auxin-induced gene in roots. CML38 (At1g76650) is involved in Ca^2+^ signaling and important for Ca^2+^-mediated developmental and stress responses and epidermal development or morphology [76]. The plastid-localized CCL protein (At3g26740) is controlled by the circadian clock during the day [77].

Only ten stress- and defense-related genes are up-regulated six days after co-cultivation. Among them are *ALCOHOL DEHYDROGENASE1* (*ADH1*), which is up-regulated in roots by osmotic stress [78] and ABA [79], the ethylene-responsive transcription factor gene *ERF105* (At5g51190) which responds to chitin treatment [80], and *INDOLE GLUCOSINOLATE O-METHYLTRANSFERASE1* (At1g21100) involved in hydroxylation reactions of the glucosinolate indole ring [81]. The *L-ascorbat oxidase At4g39830* gene is inducible by pathogens [82] and MILDEW RESISTANCE LOCUS6 mediates defense response to fungi and cell death [83]. Genes related to developmental processes code for the AAA-ATPase (At5g40010) which participates in plastidial transport [84], for the CAFFEOYL-COA 3-O-METHYLTRANSFERASE (At1g67980) which catalyses lignin monomer biosynthesis [85], and the CATION/H^+^ EXCHANGER17 (At4g23700) which regulates cation and pH homeostasis [86].

The group of common genes which are regulated at both time points includes the NAC domain transcription factor gene *JUNGBRUNNEN1* which is induced by H_2_O_2_ [87], GDSL LIPASE1 (At5g40990) that plays an important role in plant immunity [88], ERD11 (At1g02930) and the GLUTHATIONE S-TRANSFERASE TAU10 (At1g74590) which are induced by oxidative stress and bacterial infections (TAIR homepage). *ACIREDUCTONE DIOXYGENASE3* (At2g26400) which functions in H_2_O_2_ and SA signaling, is induced by hypoxia and involved in systemic acquired resistance (TAIR homepage). The oxidoreductase *At4g10500* gene is induced strongly when Arabidopsis seedlings are grown on a *P. indica* lawn [9]. Also At5g38900 (DSBA oxidoreductase) and At2g18690 have been described to be involved in defense against pathogenic fungi. All these genes are stronger up-regulated in Arabidopsis roots before a physical contact has been established between the two symbionts, which suggest that they are induced by *P. indica*-released chemical mediators.

Comparison of transcripts in rice roots, which were colonized by AM Glomalean fungi with those colonized by pathogens (*Magnaporthe grisea* and *Fusarium moniliforme*) showed that over 40% of the genes were differentially regulated by both the symbiotic and at least one of the pathogenic microbes. Güimil et al. [89] proposed that the common genes may play a role in compatibility. Furthermore, 34% of the mycorrhiza-associated rice genes were also associated with mycorrhiza in dicots, revealing a conserved pattern of response between the two angiosperm classes. Campos-Soriano and Segundo [90] hypothesized that increased demands for sugars by the fungus might be responsible for the activation of the host defense responses which will then contribute to the stabilization of root colonization by the AM fungus. However, the precise role of defense responses in mutualistic interactions is not clear. Excess root colonization might change a mutualistic association into a parasitic association (cf. [31]). This argues in favor of a role of plant defense compounds in restricting root colonization, thereby stabilizing the symbiotic interaction. Studies with the *P. indica*/Arabidopsis symbiosis support the idea [16,91]. However, inoculation with *G. intraradices* stimulated growth and biomass production in WT rice plants and plants overexpressing defense genes. The fungus activates basal defense response in mycorrhizal rice roots, including PR proteins and antioxidant enzymes. Although constitutive expression of defense genes occurred in the roots of the overexpressor lines, the symbiotic efficiency of *G. intraradices* in these plants was not affected. These results suggest that AM fungi have evolved the capacity to circumvent defense mechanisms that are controlled by the plant’s immune system [92]. Similar observations have been described for the *P. indica*/Arabidopsis interaction [93]. The authors demonstrate that a broad-spectrum suppression of innate immunity is required for colonization of Arabidopsis roots by *P. indica*.

## Conclusions

**Figure 7:**
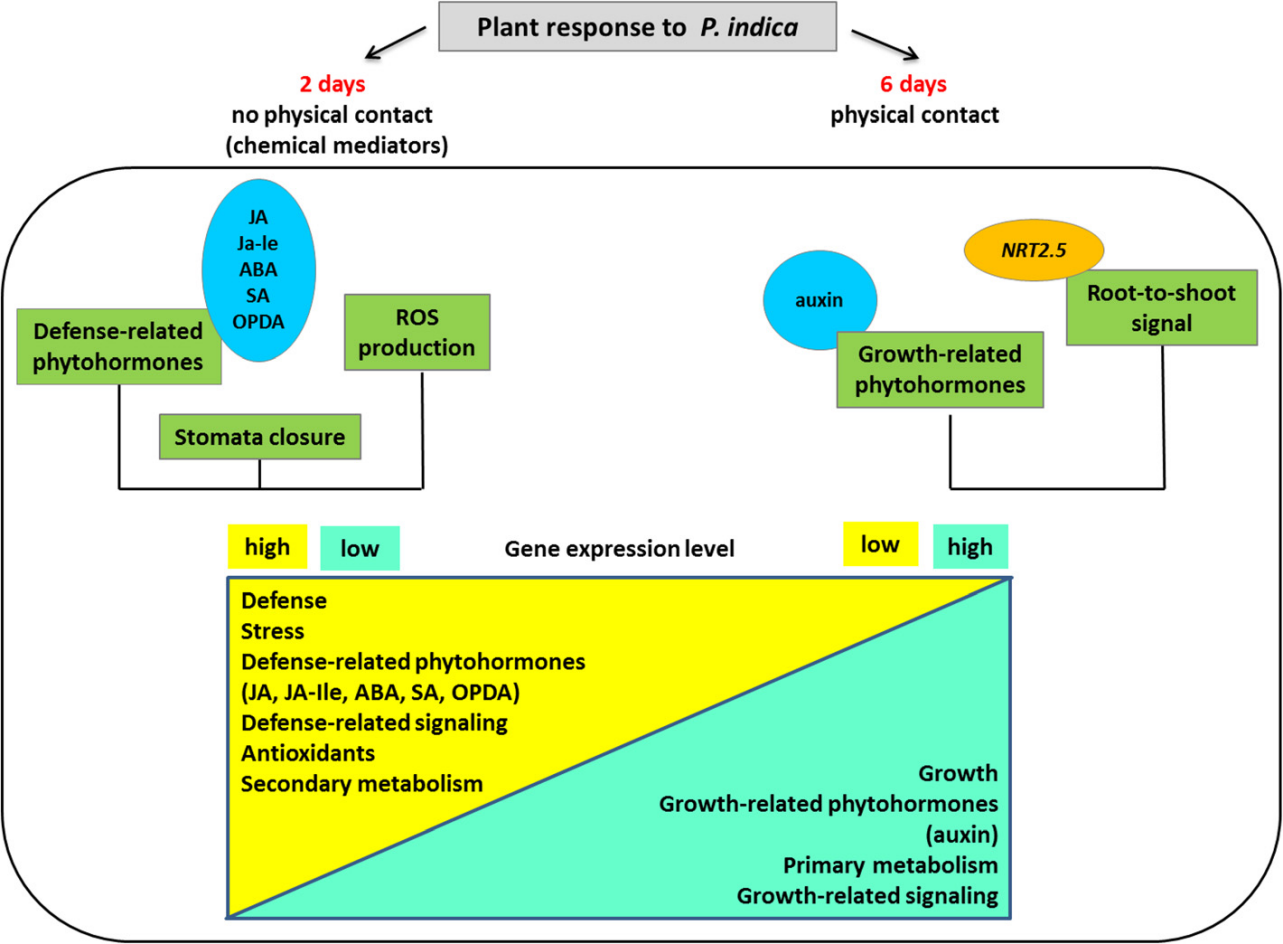
***A. thaliana*** **transcriptome changes after 2 (no physical contact between plant and fungus) and 6 (physical contact is established) days of co-cultivation with** ***P. indica*** **.**

In conclusion, our data (Figure 7) suggest that *P. indica* releases chemical compounds prior to a physical contact which activate stress and defense processes in the host (2 days). Apparently, pre-contact signaling molecules prepare the plant for the symbiotic interaction, and activation of defense may be the first line of recognition. The plant response is not restricted to roots, but also detectable in the leaves. Once root colonization has taken place (6 days) defense responses are down-regulated and genes involved in promoting plant growth, metabolism and performance are activated.

## Methods

### Growth conditions of *A. thaliana* and fungi

*A. thaliana* WT (ecotype Columbia-0) seeds were surface-sterilized and placed on Petri dishes containing MS nutrient medium [94]. After cold treatment at 4°C for 48 h, plates were incubated for 10 days at 22°C under continuous illumination (65 μmol m^−2^ sec^−1^). *P. indica* was cultured for three weeks at 22-24°C on *Aspergillus*-minimal medium [95,96 (Section A)].

### Co-cultivation of seedlings with *P. indica*

Twelve day-old (48 h cold treatment and 10 days of illumination) Arabidopsis seedlings of equal sizes were selected for co-cultivation experiments. They were transferred to PNM plates with a nylon membrane on the top [96 (Section C-Method 1)] and exposed to a fungal plug 5 mm in diameter or a KM plug of the same size without fungal hyphae (control). The plugs were placed 3 cm away from the closest root part (Additional file 1: Figure S1A). The experimental setup was identical on the split Petri dishes, except that the fungal (or control) plaques were placed on one side and the seedlings on the other side of the Petri dish. The light intensity (80 ± 5 μmol m^−2^ sec^−1^) was checked every third day to ensure that both *P. indica-* and mock-treated seedlings receive equal amounts of light.

### Gene expression

Total RNA was isolated separately from roots and shoots of WT seedlings after two and six days of co-cultivation (or mock-treatment) with *P. indica* using RNeasy Plant Mini Kit (Qiagen). After reverse-transcription, cDNA was synthesized from 1 μg total RNA using the Omniscript RT Kit (Qiagen) and oligo (dT)20 in 20 μl reaction volume. Real-time quantitative PCR was performed with gene-specific primers (Additional file 1: Table S3) and performed using the CFX connect Real-time system and the CFX manager software version 3.1 (Bio-Rad). For the amplification of the PCR products, iQ SYBR Supermix (Bio-Rad) was used according to the manufacturer’s instructions in a final volume of 20 μl. The iCycler was programmed to 95°C 2 min, 35 × (95°C 30 s, 55°C 40 s, 72°C 45 s), 72°C 10 min followed by a melting curve (55-95°C in increasing steps of 0.5°C). All reactions were repeated twice. The mRNA levels for each cDNA probe were normalized with respect to the *GAPC2* message levels. Fold induction values were calculated with the ΔΔCP equation of Pfaffl [97]. The ratio of a target gene was calculated in the *P. indica*-treated sample *versus* the mock-treated control in comparison to the *GAPC2* reference gene.

### Microarray analyses, data processing

Microarray hybridizations for *P. indica*-exposed and mock-treated Arabidopsis roots were performed with the Arabidopsis Genome Array ATH1 (Affymetrix, USA) at the Kompetenzzentrum für Fluoreszente Bioanalytik, Regensburg, Germany. The hybridization signal data were analyzed with ROBIN (http://mapman.gabipd.org/web/guest/robin-download) and MapMan (http://mapman.gabipd.org/web/guest/robin-download) programs. Statistical analysis for *t*-test and subsequent calculation of false discovery rate were performed according to ROBIN program. The microarray data given in the Supplementary Material are based on 3 biological independent experiments. The results have been submitted to GEO (http://www.ncbi.nlm.nih.gov/geo, submission number GSE58771). The *NRT2.*5 data shown here are based on Real-time PCR, since the gene was not present on all microarray chips.

Visualization of the cellular pathways and functional categories of the expression data of Arabidopsis roots after two and six days of co-cultivation with *P. indica* was carried out using the MapMan and Pegman package according to Ath_AFFY_ATH1_TAIR8_Jan 2010 (http://mapman.gabipd.org) [98]. The visualization Mapman tool was used to identify similarities and differences of different pathways involved in biotic and abiotic stress responses [98]. Wilcoxon test was used to visualize significantly expressed genes in Pegman. Venn diagrams were calculated using the expression log values of Mapman package [99]. Specifically expressed genes were determined by Venn diagram with a 3-fold change threshold. Also differentially regulated gene patterns were considered by Venn diagram according to comparative analysis of microarrays in the GEO microarray and NASC data sets.

### Microscopy of roots and stomata staining

The roots of Arabidopsis seedlings exposed to *P. indica* for two or six days were stained with trypan blue and the colonization was analysed by light and fluorescent microscopy as described in Vahabi et al. [100]. Hyphae and spores in the roots could only be detected six days after co-cultivation of the two partners (Additional file 1: Figure S1B, S2C, D1, D2). For stomata staining, detached Arabidopsis leaves were stained using 1 ml calcoflour staining solution (10 mM calcoflour in 50% glycerol, 100 μm Tween 20) for 5 min, and the epidermal layers were analysed under a light and fluorescent microscope (450–520 nm). Opened and closed stomata from 5 areas in 10 leaves from different seedlings were counted. The data are averages of three independent biological experiments. Stomata are considered as closed when no open space can be seen between the two guard cells (Figure 1).

### H_2_O_2_ and ROS measurements

Arabidopsis seedlings co-cultivated with *P. indica* for two and six days were stained with 3,3′-diaminobenzidine (DAB) as described by Daudi et al. [101]. As a result of staining a brown precipitate upon oxidation was formed, which is insoluble in aqueous and organic solvents [102,103]. For the detection/quantification of H_2_O_2_ inside the plant material, 100 mg of stained tissue was washed with acetone three times, ground to a fine powder and - after drying - dissolved in 1 ml DMSO at 90°C for 1 h. The supernatant was separated from the precipitate by centrifugation at 10,000 rpm for 5 min and was further used for spectrophotometric measurements at 270 nm (Perkin Elmer, Lambda 12) as described by Greenfield et al. [104]. The poly-DAB concentration of the plant tissue was correlated to the H_2_O_2_ concentration using a standard curve which was generated by the application of four different concentrations of H_2_O_2_ (0.1, 1, 10, 100 μg).

### Phytohormone measurement

100 mg of leaf material was frozen in liquid nitrogen and kept at -80°C. After grinding with mortar and pestle, the leaf material was extracted with 1,2 ml of methanol containing 24 ng of 9,10-D_2_-9,10-dihydrojasmonic acid, 24 ng D_4_-salicylic acid (Sigma-Aldrich, Germany), 24 ng D_6_-abscisic acid (Santa Cruz Biotechnology, Santa Cruz, USA), and 4,8 ng of JA-^13^C_6_-Ile conjugate as internal standards. JA-^13^C_6_-Ile conjugate was synthesized as described by Kramell et al. [105] using ^13^C_6_-Ile (Sigma-Aldrich, Germany). The homogenate was mixed for 30 min and centrifuged at 14,000 rpm for 20 min at 4°C. The supernatant was collected. The homogenate was re-extracted with 500 μl methanol, mixed well, centrifuged and supernatants were pooled. The combined extracts were evaporated in a speed-vac at 30°C and re-dissolved in 250 μl methanol. Chromatography was performed on an Agilent 1200 HPLC system (Agilent Technologies). Separation was achieved on a Zorbax Eclipse XDB-C18 column (50 x 4.6 mm; 1.8 μm; Agilent). Formic acid (0.05%) in water and acetonitrile were employed as mobile phases A and B, respectively. The elution profile was: 0-0.5 min, 5% B; 0.5-9.5 min, 5-42% B; 9.5-9.51 min 42-100% B; 9.51-12 min 100% B and 12.1-15 min 5% B. The mobile phase flow rate was 1.1 ml/min. The column temperature was maintained at 25°C. An API 3200 tandem mass spectrometer (Applied Biosystems) equipped with a Turbospray ion source was operated in negative ionization mode. The instrument parameters were optimized by infusion experiments with pure standards, where available. The ionspray voltage was maintained at −4500 eV. The turbo gas temperature was set at 700°C. Nebulizing gas was set at 60 psi, curtain gas at 25 psi, heating gas at 60 psi and collision gas at 7 psi. Multiple reaction monitoring (MRM) was used to monitor analyte parent ion → product ion: m/z 136.9 → 93.0 [collision energy (CE) - 22 V; declustering potential (DP) - 35 V] for SA; m/z 140.9 → 97.0 (CE - 22 V; DP - 35 V) for D4-SA; m/z 209.1 → 59.0 (CE - 24 V; DP - 35 V) for JA; m/z 213.1 → 56.0 (CE - 24 V; DP - 35 V) for 9,10-D2-9,10-dihydrojasmonic acid; m/z 263.0 → 153.2 (CE - 22 V; DP - 35 V) for ABA; m/z 269.0 → 159.2 (CE - 22 V; DP - 35 V) for D6-ABA; m/z 322.2 → 130.1 (CE - 30 V; DP - 50 V) for JA-Ile conjugate; m/z 328.2 → 136.1 (CE - 30 V; DP - 50 V) for JA-^13^C_6_-Ile conjugate. Both Q1 and Q3 quadrupoles were maintained at unit resolution. Analyst 1.5 software (Applied Biosystems) was used for data acquisition and processing. Linearity in ionization efficiencies were verified by analyzing dilution series of standard mixtures. Phytohormones were quantified relative to the signal of their corresponding internal standard. For quantification of 12-oxophytodienoic acid, *cis*-OPDA, 9,10-D_2_-9,10-dihydro-JA was used as the internal standard applying an experimentally determined response factor of 1.

### Availability of supporting data

All the supporting data are included as Additional file 1.

## Additional file

Additional file 1: Figure S1. Experimental design for the co-cultivation assays. **Figure S2**. Localization of *P. indica* mycelium and spores in and around Arabidopsis roots. **Table S1**. MAPMAN analysis of the genes which are regulated at least 3-fold in the roots of seedlings exposed to *P. indica* for two or six days. **Table S2**. Regulated genes in *A. thaliana* roots after two days interaction with *P. indica* grown on normal or split Petri dishes. **Table S3**. List of primers for RT-PCR used in this study.

## Abbreviations

*Pi*: *Piriformospora indica*
ROS: Reactive oxygen species
MAMP: Microbe-associated molecular pattern
AM: Arbuscular mycorrhiza
JA: Jasmonic acid
SA: Salicylic acid
ABA: Abscisic acid
OPDA: 12-oxo-phytodienoic acid
JA-Ile: Jasmonic acid-isoleucine
WT: Wild-type
