# Supplementary material for: The interaction of Arabidopsis with Piriformospora indica shifts from initial transient stress induced by fungus-released chemical mediators to a mutualistic interaction after physical contact of the two symbionts
Source: BMC Plant Biol. 2015 Feb 21;15:58. doi: 10.1186/s12870-015-0419-3 (PMC4384353; doi:10.1186/s12870-015-0419-3)
Supplement: Additional file 1: Figure S1. — Experimental design for the co-cultivation assays. Figure S2. Localization of P. indica mycelium and spores in and around Arabidopsis roots. Table S1. MAPMAN analysis of the genes which are regulated at least 3-fold in the roots of seedlings exposed to P. indica for two or six days. Table S2. Regulated genes in A. thaliana roots after two days interaction with P. indica grown on normal or split Petri dishes. Table S3. List of primers for RT-PCR used in this study. [file 12870_2015_419_MOESM1_ESM.pdf]

The interaction of Arabidopsis with *Piriformospora indica* shifts from initial transient stress induced by fungus-released chemical mediators to a mutualistic interaction after physical contact of the two symbionts

Khabat Vahabi[1], Irena Sherameti[1], Madhunita Bakshi[1], Anna Mrozinska[1], Anatoli Ludwig[1], Michael Reichelt[2], Ralf Oelmüller[1*]

[1]Institute of General Botany and Plant Physiology, Friedrich-Schiller-University Jena, Dornburger Str. 159, 07743 Jena, Germany

[2]Max-Planck Institute for Chemical Ecology, Hans-Knöll-Straße 8, 07745 Jena, Germany

(1) Khabat Vahabi
E-mail: khabat.v@gmail.com

(2) Irena Sherameti
E-mail: irena.sherameti@uni-jena.de

(3) Madhunita Bakshi
E-mail: bakshi.madhunita@gmail.com

(4) Anna Mrozinska
E-mail: amrozinska@ice.mpg.de

(5) Anatoli Ludwig
E-mail: aludwig82@gmail.com

(6) Michael Reichelt
E-mail: reichelt@ice.mpg.de

(7)***Correspondence:** Ralf Oelmüller

E-mail: b7oera@uni-jena.de

Tel.: +49 3641 949231

Fax: +49 3641 949232

Running title: *P. indica*´s interaction with Arabidopsis

Additional File 1

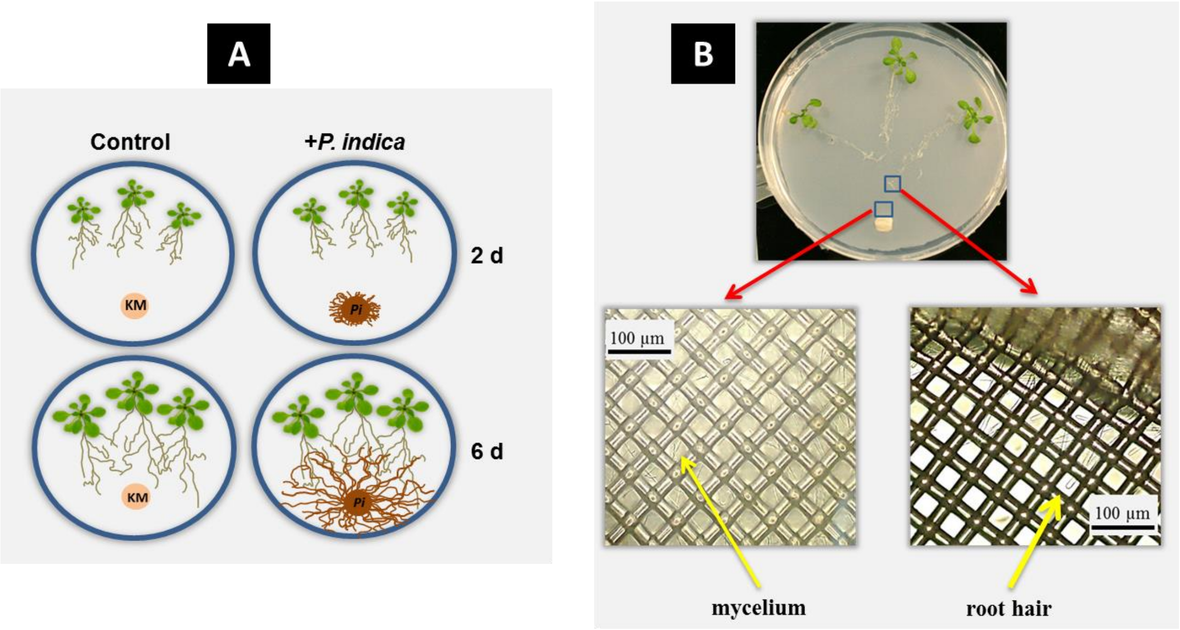

Figure S1. Experimental design for the co-cultivation assays. **(A)** Twelve day-old (48 h cold-treatment and 10 days of illumination) Arabidopsis seedlings were transferred to a nylon membrane on top of PNM medium in a Petri dish. A fungal plaque with *P. indica* which was grown on Kaefer medium (*Pi*), or without *P. indica* (KM) was posed 3 cm away from the closest root material. *A. thaliana* seedlings with *Pi*/KM treatment were cultivated for two or six days before analyses. (**B)** The position of the fungal hyphae and root hairs can easily be distinguished on the nylon net.

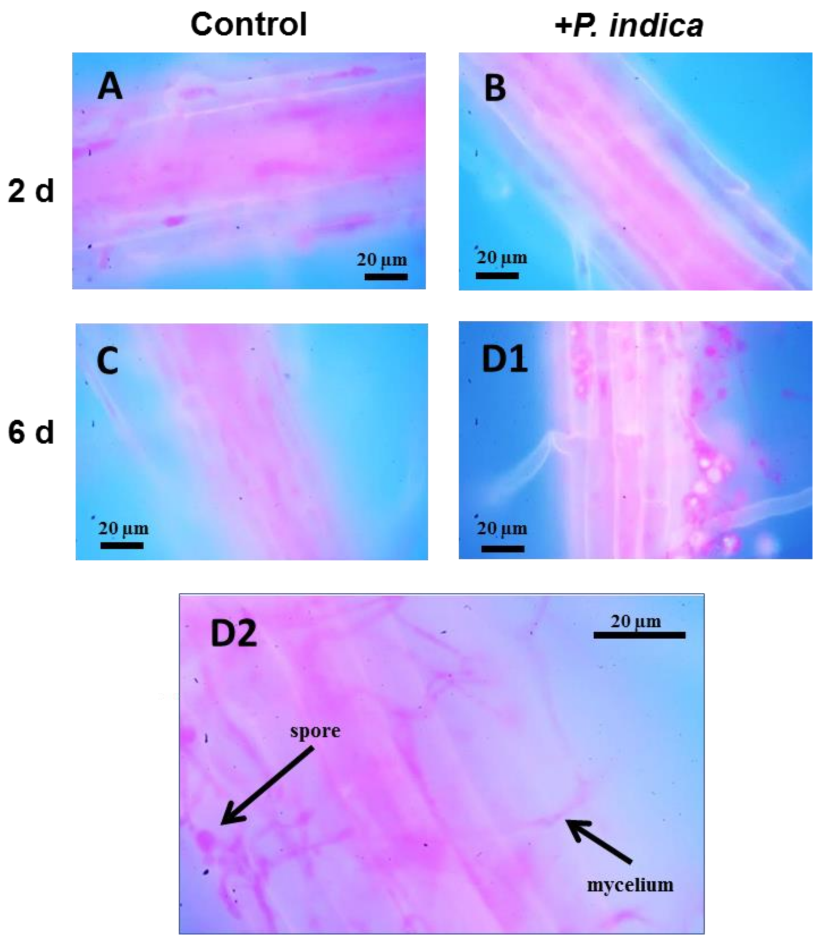

Figure S2. Localization of *P. indica* mycelium and spores in and around Arabidopsis roots. The roots of Arabidopsis seedlings exposed to *P. indica* for two or six days were stained with trypan blue and the spores and mycelium were detected by fluorescent microscopy. No fungal material can be detected in the control (**A** and **C**) as well as two days after exposure to *P. indica* (**B**). After the co-cultivation for six days, spores and mycelium are visible (**D1** and **D2** with different magnifications).

Table S1. MAPMAN analysis of the genes which are regulated at least 3-fold in the roots of seedlings exposed to *P. indica* for two or six days. Two days: genes which are only regulated two days after co-cultivation. Six days: genes which are only regulated six days after co-cultivation. [2 and 6] days: common genes which are regulated at both time points. Regulation was determined relative to the control (agar plaque without fungal hyphae). The microarray data for seedlings at t = 0 and seedlings exposed to the agar plug without hyphae for two or six days were almost identical. Fold-regulation is given as log10 values. The results show the average of three independent biological experiments with 2-3 technical RT-PCR reactions.

(A) Genes up- or down-regulated only at two days after co-cultivation. **(B)** Common genes up- or down-regulated at [2 and 6] days after co-cultivation. **(C)** Genes up- or down-regulated only at six days after co-cultivation. **(D)** 33 genes were randomly chosen and their regulation was tested by Real-time PCR analysis.

Table S1A. Genes up- or down-regulated only at two days after co-cultivation.

Nr.	BinName	AGI	Description	2 d + *P. indica*
**1**	aminoacid metabolism, synthesis	At5g05730	ASA1 (anthranilate synthase subunit 1)	1.11
**2**	development, unspecified	At2g38860	YLS5	1.03
**3**	development, unspecified	At4g35770	SEN1 (SENESCENCE 1)	-1.66
**4**	DNA synthesis/chromatin structure	At2g18050	HIS1-3 (HISTONE H1-3)	-1.77
**5**	hormone metabolism, auxin induced	At1g59500	GH3.4 (indole-3-acetic acid amido synthetase)	1.22
**6**	hormone metabolism, ethylene induced, co-factor and vitamine metabolism	At2g38230	PDX1.1 (Pyridoxine biosynthesis 1.1)	-1.43
**7**	minor CHO metabolism, raffinose family, galactinol synthases, putative	At1g60470	GolS4 Arabidopsis thaliana (Galactinol Synthase 4, transferase)	1.52
**8**	cytochrome P450	At2g24180	CYP71B6 (CYTOCHROME P450 71B6, electron carrier/heme binding/iron ion binding/monooxygenase/oxygen binding)	1.25
**9**	cytochrome P450	At3g26830	PAD3 (PHYTOALEXIN DEFICIENT 3, dihydrocamalexic acid decarboxylase/monooxygenase/oxygen binding)	1.65
**10**	cytochrome P450	At4g37370	CYP81D8 (electron carrier/heme binding/iron ion binding/monooxygenase/oxygen binding)	1.45
**11**	glutathione S transferases	At2g02930	ATGSTF3 (GLUTATHIONE S-TRANSFERASE F3)	1.22
**12**	glutathione S transferases	At5g02780	In2-1 protein, putative	1.45
**13**	nitrilases	At2g30670	tropinone reductase, putative	2.80
**14**	plastocyanin-like	At5g20230	BCB BLUE-COPPER-BINDING PROTEIN, copper ion binding/electron carrier	1.10
**15**	protease inhibitor/seed storage/lipid transfer protein LTP	At4g12550	AIR1 (lipid binding)	-1.33
**16**	UDP glucosyl and glucoronyl transferases	At2g16890	UDP-glucoronosyl/UDP-glucosyl transferase	-1.46
**17**	UDP glucosyl and glucoronyl transferases	At3g21560	UGT84A2, UDP-glycosyltransferase/sinapate 1-glucosyltransferase	-1.18
**18**	UDP glucosyl and glucoronyl transferases	At3g53150	UGT73D1, UDP-glucosyl transferase 73D1	1.60
**19**	protein degradation, cysteine protease	At4g36880	CP1 (CYSTEINE PROTEINASE1, cysteine-type endopeptidase)	1.94
**20**	protein degradation, subtilases	At1g32940	SBT3.5 (identical protein binding/serine-type endopeptidase)	1.50
**21**	protein postranslational modification	At2g34180	CIPK13 (CBL-INTERACTING PROTEIN KINASE 13, ATP binding/kinase/protein kinase)	1.20
**22**	protein synthesis, ribosomal protein, eukaryotic 60S subunit L34	At3g01175	unknown function	1.35
**23**	redox glutaredoxins	At1g28480	GRX480 (electron carrier/protein disulfide oxidoreductase)	1.14
**24**	RNA regulation of transcription, MYB domain transcription factor family, secondary metabolism	At1g18570	MYB51 (MYB DOMAIN PROTEIN 51, DNA binding/transcription factor)	1.38
**25**	RNA regulation of transcription, unclassified	At3g59080	aspartyl protease family protein	1.14

**26**	RNA regulation of transcription, WRKY domain transcription factor family	At2g40750	WRKY54 (transcription factor)	2.41
**27**	RNA regulation of transcription, WRKY domain transcription factor family	At3g56400	WRKY70 (transcription factor/transcription repressor)	2.12
**28**	secondary metabolism, flavonoids, anthocyanins	At2g22590	transferase, transferring glycosyl groups | chr2	-1.87
**29**	calcium signaling	At1g76650	CML38 (calcium-binding EF hand family protein)	-1.14
**30**	calcium signaling	At2g41100	TCH3 (TOUCH 3, calcium ion binding)	1.59
**31**	calcium signaling	At3g63380	ACA12 (calcium-transporting ATPase, putative)	1.60
**32**	calcium signaling	At4g33050	EDA39 (embryo sac development arrest 39, calmodulin binding)	1.57
**33**	light signaling	At3g26740	CCL (CCR-LIKE)	-1.17
**34**	light signaling	At4g14690	ELIP2 (EARLY LIGHT-INDUCIBLE PROTEIN 2, chlorophyll binding)	-1.71
**35**	signaling, receptor kinases	At1g51890	leucine-rich repeat protein kinase, putative	1.02
**36**	abiotic stress, unspecified	At1g18970	*GLP4* (GERMIN-LIKE PROTEIN, manganese ion binding)	1.49
**37**	abiotic stress, unspecified	At1g18980	germin-like protein	1.20
**38**	biotic stress	At2g38870	protease inhibitor	1.00
**39**	biotic stress	At2g43590	chitinase	1.55
**40**	biotic stress, PR-proteins	At3g50950	disease resistance protein CC-NBS-LRR class	1.13
**41**	not assigned, no ontology	At4g20000	VQ motif-containing protein	1.40
**42**	not assigned, no ontology	At5g42010	WD-40 repeat family protein	1.07
**43**	not assigned, unknown	At1g19530	unknown protein	-1.71
**44**	not assigned, unknown	At1g36640	unknown protein	1.67
**45**	not assigned, unknown	At1g72060	serine-type endopeptidase inhibitor	-1.30
**46**	not assigned, unknown	At3g56290	unknown protein	-1.05
**47**	not assigned, unknown	At4g04990	unknown protein	1.31
**48**	not assigned, unknown	At5g22270	unknown protein	1.58

Table S1B. Common genes up- or down-regulated at [2 and 6] days after co-cultivation.

Nr.	BinName	AGI	Description	2 d + *P. indica*	6 d + *P. indica*
**1**	cell wall, HRGP	At1g26240	proline-rich extensin-like family protein	2.86	1.6
**2**	cell organisation	At1g10340	ankyrin repeat family protein	1.12	1.09
**3**	cell organisation	At4g14365	C3HC4-type RING finger family protein/ankyrin repeat family protein	1.83	1.04
**4**	development, storage proteins	At2g26560	PLA2A PHOSPHOLIPASE A 2A, lipase/ nutrient reservoir	3.19	1.13
**5**	development, unspecified	At2g43000	anac042 Arabidopsis NAC domain containing protein 42, transcription factor	2.84	1.25
**6**	hormone metabolism, jasmonate.synthesis-degradation, 12-Oxo-PDA-reductase	At1g18020	12-oxophytodienoate reductase	1.39	1.36
**7**	lipid metabolism, lipid degradation	At2g39400	hydrolase, alpha/beta fold family protein	1.13	1.44
**8**	GDSL-motif lipase	At5g40990	GLIP1 GDSL LIPASE1, carboxylesterase/lipase	3.66	1.45
**9**	glutathione S transferases	At1g02930	ATGSTF6, GST1, ERD11, ATGSTF3, GSTF6 | ATGSTF6 GLUTATHIONE S-TRANSFERASE, copper ion binding / glutathione binding/glutathione transferase	2.89	1.06
**10**	glutathione S transferases	At1g14540	anionic peroxidase	2.05	1.13
**11**	glutathione S transferases	At1g14550	anionic peroxidase	1.97	1.52
**12**	glutathione S transferases	At1g74590	GSTU10 GLUTATHIONE S-TRANSFERASE TAU 10, glutathione transferase	2.07	1.02
**13**	nitrilases, nitrile lyases, berberine bridge enzymes, reticuline oxidases, troponine reductases	At1g26380	FAD-binding domain-containing protein	4.27	1.64
**14**	nitrilases, *nitrile lyases, berberine bridge enzymes, reticuline oxidases, troponine reductases	At1g26390	FAD-binding domain-containing protein	4.97	2.15
**15**	O-methyl transferases	At1g21110	O-methyltransferase	1.63	1.5
**16**	O-methyl transferases	At1g21120	O-methyltransferase	2.43	1.76
**17**	O-methyl transferases	At1g21130	O-methyltransferase	2	1.8
**18**	peroxidases	At3g03670	peroxidase	1.05	1.77
**19**	peroxidases	At5g39580	peroxidase	3.25	1.61
**20**	protein degradation, ubiquitin, E3, RING	At3g61390	U-box domain-containing protein	1.07	1.26
**21**	secondary metabolism, phenylpropanoids	At5g42830	transferase family protein	1.98	1.09
**22**	secondary metabolism, sulfur-containing glucosinolates, cytochrome P450 monooxygenase	At5g57220	CYP81F2; electron carrier/ heme binding/iron ion binding /monooxygenase/oxygen binding	4.23	1.82
**23**	calcium signaling	At5g26920	CBP60G CAM-BINDING PROTEIN 60-LIKE.G, calmodulin binding	4.04	1.72
**24**	calcium signaling	At5g39670	calcium-binding EF hand family protein	1.54	1.26

**25**	light signaling	At3g22840	ELIP1 EARLY LIGHT-INDUCABLE PROTEIN, chlorophyll binding	-2.37	-1
**26**	signaling, MAP kinases	At1g01560	ATMPK11; MAP kinase/ kinase	1.27	1.57
**27**	abiotic stress, unspecified	At4g14630	GLP9 GERMIN-LIKE PROTEIN 9, manganese ion binding/nutrient reservoir	1.92	1.76
**28**	biotic stress	At2g15120	pseudogene, disease-resistance family protein/fatty acid elongase-related	2.36	1.22
**29**	biotic stress	At2g43570	chitinase	2.61	1.68
**30**	biotic stress, PR-proteins, plant defensins	At2g43510	ATTI1, serine-type endopeptidase inhibitor	2.23	1.03
**31**	biotic stress, signalling	At5g64905	PROPEP3 Elicitor peptide 3 precursor	1.7	1.26
**32**	not assigned, no ontology	At2g26400	ATARD3 ACIREDUCTONE DIOXYGENASE 3, acireductone dioxygenase [ironII)-requiring]	1.67	1.41
**33**	not assigned, no ontology	At4g10500	oxidoreductase	2.82	1.73
**34**	not assigned, no ontology	At5g38900	DSBA oxidoreductase family protein	2.57	1.18
**35**	not assigned, no ontology, glycine rich protein	At4g37900	glycine-rich protein	1.04	1.25
**36**	not assigned, unknown	At1g22890	unknown protein	1.68	1.27
**37**	not assigned, unknown	At2g18690	unknown protein	1.13	1.02
**38**	not assigned, unknown	At3g18250	unknown protein	2.04	1.64
**39**	not assigned, unknown	At4g28460	unknown protein	2.7	2.04
**40**	not assigned, unknown	At4g37290	unknown protein	2.54	1.2
**41**	not assigned, unknown	At1g26250	proline-rich extensin protein	1.03	1.29

Table S1C. Genes up- or down-regulated only at six days after co-cultivation.

Nr.	BinName	AGI	Description	6 d + *P. indica*
**1**	glycolysis, cytosolic branch	At5g04120	phosphoglycerate/bisphosphoglycerate mutase family protein	-1.16
**2**	fermentation	At1g77120	ADH1 ALCOHOL DEHYDROGENASE 1	1.58
**3**	cytochrome P450	At4g31940	CYP82C4; electron carrier/heme binding/iron ion binding monooxygenase/oxygen binding	-1.04
**4**	O-methyl transferases	At1g21100	O-methyltransferase	1.24
**5**	metal transport	At4g23700	ATCHX17 CATION/H^+ EXCHANGER 17	1.16
**6**	transport membrane system, unknown	At4g25220	transporter	-1.15
**7**	secondary metabolism, phenylpropanoids, lignin biosynthesis, CCoAOMT	At1g67980	CCoAMT; caffeoyl-CoA O-methyltransferase	1.22
**8**	hormone metabolism, ethylene signal transduction	At5g51190	AP2 domain-containing transcription factor	1.08
**9**	biotic stress, signaling, MLO-like	At1g61560	MLO6 MILDEW RESISTANCE LOCUS O, calmodulin binding	1.03
**10**	redox ascorbate and glutathione ascorbate	At4g39830	L-ascorbate oxidase	1.36
**11**	protein degradation, AAA type	At5g40010	AATP1 AAA-ATPase 1	1.15
**12**	receptor kinases signaling, S-locus glycoprotein like	At1g61500	S-locus protein kinase	1.25
**13**	not assigned, unknown	At4g04630	unknown protein	1

Table S1D. 33 genes were randomly chosen and their regulation was tested by Real-time PCR analysis.

Nr.	Gene	Gene name	2 d + *P.indica* (Real-Time PCR)	2 d + *P. indica* (microarray)	6 d + *P. indica* (Real-Time PCR)	6 d + *P. indica* (microarray)
**1**	At1g42970	GAPB	-0.64	-0.43	0.42	0.22
**2**	At4g38970	fructose-bisphosphate aldolase	-0.77	-0.56	0.20	0.29
**3**	At1g20190	ATEXPA11	0.71	0.02	0.23	0.13
**4**	At4g25700	BETA-OHASE 1	-0.80	-0.29	0.07	0.11
**5**	At5g57220	CYP81F2	3.80	4.23	0.11	1.82
**6**	At1g18570	MYB51	1.81	1.38	0.81	0.94
**7**	At5g51190	AP2 domain-containing transcription factor	-0.78	-0.32	1.57	1.08
**8**	At2g43590	chitinase	2.25	1.55	1.30	0.97
**9**	At2g43510	ATTI1; serine-type endopeptidase inhibitor	1.55	2.23	1.17	1.03
**10**	At5g38910	germin-like protein	4.81	6.53	2.06	2.42
**11**	At1g28480	GRX480	1.57	1.14	0.80	0.88
**12**	At1g26390	FAD-binding domain-containing protein	5.35	4.97	2.75	2.15
**13**	At1g02930	ATGSTF6	3.16	2.89	1.46	1.06
**14**	At4g31940	CYP82C4	0.88	0.98	-1.26	-1.04
**15**	At5g57220	CYP81F2	3.51	4.23	2.73	1.82
**16**	At5g39580	peroxidase	4.01	3.25	1.24	1.61
**17**	At1g18570	MYB51	2.03	1.38	0.65	0.94
**18**	At2g40750	WRKY54	2.29	2.41	0.10	0.26
**19**	At1g61500	S-locus protein kinase	0.62	0.58	1.80	1.25
**20**	At1g76650	CML38	-1.01	-1.14	0.41	0.72
**21**	At5g26920	CBP60G	3.01	4.04	2.04	1.72
**22**	At4g14690	ELIP2	-2.15	-1.71	-0.40	-0.75
**23**	At3g60270	Cupredoxin superfamily protein	4.27	3.40	2.29	1.41
**24**	At4g10500	oxidoreductase, 2OG-Fe(II) oxygenase family protein	2.37	2.82	0.92	1.73
**25**	At5g37510	EMB	0.30	0.00	0.03	-0.22
**26**	At2g40890	CYP89	0.27	0.42	0.00	0.03
**27**	At3g48730	GSA	0.57	0.14	-0.23	0.01
**28**	At1g03630	PORC	-0.23	0.13	0.40	-0.02
**29**	At3g04720	PR4	0.00	0.54	-1.86	-0.02
**30**	At3g12500	PR3	1.36	1.02	3.49	-0.02
**31**	At5g44420	PDF1.2	0.47	-1.07	-1.43	-0.32
**32**	At2g43790	MPK6	0.10	0.02	-0.86	0.00
**33**	At2g38120	AUX1	0.13	-0.10	0.27	-0.15

Table S2. Regulated genes in *A. thaliana* roots after two days interaction with *P. indica* grown on normal or split Petri dishes (*PD = Petri dish).

Nr.	Gene	Gene name	2 d + *P .indica* Normal PD*	2 d + *P. indica* Split PD*
**1**	At1g42970	*GAPB*	-0.64	0.08
**2**	At4g38970	fructose-bisphosphate aldolase	-0.77	0.13
**3**	At1g20190	*EXPA11*	0.71	0.34
**4**	At4g25700	*BETA-OHASE 1*	-0.80	0.22
**5**	At5g57220	*CYP81F2*	3.80	-0.15
**6**	At1g18570	*MYB51*	1.81	0.36
**7**	At5g51190	AP2 domain-containing transcription factor	-0.78	0.12
**8**	At2g43590	chitinase	2.25	0.68
**9**	At2g43510	*ATTI1*; serine-type endopeptidase inhibitor	1.55	0.47
**10**	At5g38910	germin-like protein	4.81	-0.30
**11**	At1g28480	*GRX480*	1.57	0.08
**12**	At1g26390	FAD-binding domain-containing protein	5.35	0.60
**13**	At1g02930	*GSTF6*	3.16	-0.04
**14**	At4g31940	*CYP82C4*	0.88	-0.36
**15**	At5g57220	*CYP81F2*	3.51	0.04
**16**	At5g39580	peroxidase	4.01	0.42
**17**	At1g18570	*MYB51*	2.03	0.76
**18**	At2g40750	*WRKY54*	2.29	0.51
**19**	At1g61500	S-locus protein kinase	0.62	0.06
**20**	At1g76650	*CML38*	-1.01	0.35
**21**	At5g26920	*CBP60G*	3.01	0.12
**22**	At4g14690	*ELIP2*	-2.15	-0.07
**23**	At3g60270	Cupredoxin superfamily protein	4.27	0.64
**24**	At4g10500	oxidoreductase, 2OG-Fe(II) oxygenase family protein	2.37	0.05
**25**	At5g37510	*EMB*	0.30	0.27
**26**	At2g40890	*CYP89*	0.27	-0.30
**27**	At3g48730	*GSA*	0.57	0.15
**28**	At1g03630	*PORC*	-0.23	0.26
**29**	At3g04720	*PR4*	0.00	-0.69
**30**	At3g12500	*PR3*	1.36	-0.01
**31**	At5g44420	*PDF1.2*	0.47	0.84
**32**	At2g43790	*MPK6*	0.10	0.11
**33**	At2g38120	*AUX1*	0.13	0.48

Table S3. List of primers for RT-PCR used in this study.

Gene	Forward primer (5' - 3')	Reverse primer (5' - 3')
At1g03630	CACATACCGCTGTTTCGGCTTC	AGACTAGGGTCACTCACAACCTG
At1g42970	ACTCCTACACCGGAGACCAAAG	GCTGCGGCAACACTAATGACAC
At2g38120	CAGCTGCGCATCTAACCAAGTG	GATGAGATAAGCAGTCCAGCTTCC
At2g43790	GGGTGCTTATGGCATCGTTTGTTC	ATCGCAACGCTCTCGTTAGTTTC
At2g40890	ACAGCATTTCGTTGATGCGTTGC	CCCTGCCGTGATCATATCCCATAG
At3g48730	TGGATTCTGCAAAGGGCTCACG	TCAGCCAAAGCAGCAAGAACCTC
At3g60270	TGCAGCCTTGGCATGAAACTCG	ACGGTGGAGGCTCTAATGAAACG
At4g25700	GGCATAACGGTGTTTGGAATCGC	CTTGTCTGTGTGATGTAGCTGGTG
At4g38970	ATACGCTGCCATTTCACAGGAC	TGTCGTGTTCTCCATCCAACAAG
At5g26920	CGATAGGACCTTTGTGGGTCATCC	ACTTCCTTGAAAGTCGATGTGCTG
At5g37510	TCGGTGTATCCAGTGTACAAGGTG	CAATTTCCTCTCCACTACCACGAC
At5g39580	GCGATCTCGTCACTCTTGTTGGAG	TAAACCCACATGCAGCTGTTCCG
At5g57220	CATCATCAAAGGGCTCATGCTCAG	AATGTTACGGCCGCAGTATCCG
At1g26390	ACGCCACAATGAGTAGCCTGAG	TCACCACTCGGATTGCTTCCAAC
At1g20190	GCTTCTGGAACAATGGGTGGAG	AGCATTCTCCGCAAGAAGCTC
At1g02930	AGCCTTTCATCCTTCGCAACCC	TGTCCTTGCCAGTTGAGAGAAGG
AT1g28480	ACGGAGAGGATGTTGCATGTGTC	AATCTCAAGGACCGCCGGATTC
At1g76650	AAGAGGGAAGATTCAGCCGGAGAG	CTCTGTTCTTGTCCTCACCATTGC
At1g18570	CCTTCACGGCAACAAATGGTCTG	TACCGGAGGTTATGCCCTTGTG
At5g51190	TCTAAGCCAACGCAAACCACCTC	TTCTCTTGAACCACCGGTGCAG
At2g43510	AGGCTATCGTTTCCATCTTCGTTG	TCTCCGGCACATCGGAAATGAC
At1g61500	AGATGCAGAGTGGAACATATCGC	CCTGGGACATCTTGTGGTTTCAG
At4g14690	CCACCAGTTAGCAAGCCTAAGGTG	TGGACCGCTAAACGCTAGCAAATC
At2g40750	CACTGCTCAGAACCATGTCAATGC	TGCTGTCACACTGCTGGTGTTG
At4g31940	CAAATCTACCTGCCTGGCACTG	TGGCCCATGTAAGGGTTGATGC
At2g43590	ACTCACGAGACCGGACATTTCTGC	TGGGTATTGTGTGTTGCTGCTCTG
At5g44420	CTTGTGTGCTGGGAAGACATA	AGCACAGAAGTTGTGCGAGAA
At5g38910	TCATTAAGCAAGGCTTCGGAC	TTTGACGTGACAAACCCAACG
At3g04720	GACCTCGTGGTCAAGCTTCTT	TTGCTACATCCAAATCCAAGC
At3g12500	TCATGGGGCTACTGTTTCAAG	TATTGCTCTACCGCATAGACC
At4g10500	TATCGGCGACCAAATGCAGGTC	ACTACGGCTCTATGGAGCACAC
